# Genetic Characterization of Influenza A Viruses in Japanese Swine in 2015 to 2019

**DOI:** 10.1128/JVI.02169-19

**Published:** 2020-07-01

**Authors:** Junki Mine, Yuko Uchida, Nobuhiro Takemae, Takehiko Saito

**Affiliations:** a Division of Transboundary Diseases, National Institute of Animal Health, National Agriculture and Food Research Organization (NARO), Tsukuba, Ibaraki, Japan; b United Graduate School of Veterinary Sciences, Gifu University, Gifu, Japan; Icahn School of Medicine at Mount Sinai

**Keywords:** Japan, influenza, phylogeny, pig, reassortment

## Abstract

To assess the current status of influenza A viruses of swine (IAVs-S) throughout Japan and to investigate how these viruses persisted and evolve on pig farms, we genetically characterized IAVs-S isolated during 2015 to 2019. Nasal swab samples collected through active surveillance and lung tissue samples collected for diagnosis yielded 424 IAVs-S, comprising 78 H1N1, 331 H1N2, and 15 H3N2 viruses, from farms in 21 sampled prefectures in Japan. Phylogenetic analyses of surface genes revealed that the 1A.1 classical swine H1 lineage has evolved uniquely since the late 1970s among pig populations in Japan. During 2015 to 2019, A(H1N1)pdm09 viruses repeatedly became introduced into farms and reassorted with endemic H1N2 and H3N2 IAVs-S. H3N2 IAVs-S isolated during 2015 to 2019 formed a clade that originated from 1999–2000 human seasonal influenza viruses; this situation differs from previous reports, in which H3N2 IAVs-S derived from human seasonal influenza viruses were transmitted sporadically from humans to swine but then disappeared without becoming established within the pig population. At farms where IAVs-S were frequently isolated for at least 3 years, multiple introductions of IAVs-S with phylogenetically distinct hemagglutinin (HA) genes occurred. In addition, at one farm, IAVs-S derived from a single introduction persisted for at least 3 years and carried no mutations at the deduced antigenic sites of the hemagglutinin protein, except for one at the antigenic site (Sa). Our results extend our understanding regarding the status of IAVs-S currently circulating in Japan and how they genetically evolve at the farm level.

**IMPORTANCE** Understanding the current status of influenza A viruses of swine (IAVs-S) and their evolution at the farm level is important for controlling these pathogens. Efforts to monitor IAVs-S during 2015 to 2019 yielded H1N1, H1N2, and H3N2 viruses. H1 genes in Japanese swine formed a unique clade in the classical swine H1 lineage of 1A.1, and H3 genes originating from 1999–2000 human seasonal influenza viruses appear to have become established among Japanese swine. A(H1N1)pdm09-derived H1 genes became introduced repeatedly and reassorted with endemic IAVs-S, resulting in various combinations of surface and internal genes among pig populations in Japan. At the farm level, multiple introductions of IAVs-S with phylogenetically distinct HA sequences occurred, or IAVs-S derived from a single introduction have persisted for at least 3 years with only a single mutation at the antigenic site of the HA protein. Continued monitoring of IAVs-S is necessary to update and maximize control strategies.

## Abstract (precis)

Understanding the current status of influenza A viruses of swine (IAVs-S) and their evolution at the farm level is important for controlling these pathogens. Efforts to monitor IAVs-S during 2015 to 2019 yielded H1N1, H1N2, and H3N2 viruses. H1 genes in Japanese swine formed a unique clade in the classical swine H1 lineage of 1A.1, and H3 genes originating from 1999–2000 human seasonal influenza viruses appear to have become established among Japanese swine. A(H1N1)pdm09-derived H1 genes became introduced repeatedly and reassorted with endemic IAVs-S, resulting in various combinations of surface and internal genes among pig populations in Japan. At the farm level, multiple introductions of IAVs-S with phylogenetically distinct HA sequences occurred, or IAVs-S derived from a single introduction have persisted for at least 3 years with only a single mutation at the antigenic site of the HA protein. Continued monitoring of IAVs-S is necessary to update and maximize control strategies.

## INTRODUCTION

Influenza A virus of swine (IAV-S) is a major respiratory pathogen and causes broadly prevalent disease in pigs worldwide. IAV-S typically causes mild symptoms, such as fever, loss of appetite, dullness, and low mortality rates (1), but coinfection with IAV-S and other pathogens, such as porcine reproductive and respiratory syndrome virus, increases the mortality rate and decreases average daily weight gain (2). Thus, IAV-S, alone and in combination with other pathogens, causes tremendous economic loss for pig farmers worldwide (3, 4). Additionally, pigs are susceptible to both human and avian influenza viruses, as they express the receptors to both influenza viruses on their tracheal epithelial cells (5). With such characteristics, pigs serve as a reservoir and a “mixing vessel” in which reassortment events of influenza viruses occur to generate novel gene constellations with pandemic potential (6). Therefore, surveillance activities on IAV-S have been accelerated not only for moderating the economic loss to the swine industry all over the world but also for monitoring emerging IAVs-S with the potential to be the next pandemic.

Worldwide circulation of IAV-S changed dramatically after the pandemic caused by A(H1N1)2009 [A(H1N1)pdm09] viruses. Before 2009, three IAV-S subtypes, H1N1, H1N2, and H3N2, predominated in swine throughout the world (7). Classical swine H1N1 IAVs-S were first isolated in North America in 1930 and have been infecting pigs endemically there since the 1970s, currently circulating among pig population worldwide, whereas avian-like H1N1 IAVs-S have stably persisted in Europe since 1979 (7, 8). In Asia, avian-like H1N1 IAVs-S was first isolated in China in 1993 (7, 9, 10), and reassortant IAVs-S containing genes derived from avian-like H1N1 IAVs-S have been reported in Thailand (11–14), South Korea (15), and Vietnam (16). A/Hong Kong/1968-like H3N2 viruses have been isolated from pigs in Asia and Europe, indicating the worldwide circulation of human-like H3N2 IAVs-S, while some cases of the isolation of avian-like H3N2 IAVs-S were reported in Asia in the 1970s (17–21). In 1998, a triple-reassortant H3N2 virus emerged in North America through reassortment among a human seasonal virus, a classical swine IAV-S, and an avian influenza virus, which was followed by the emergence of H1N1 and H1N2 triple-reassortant IAVs-S and their circulation in North America. (22). Subsequently, in April 2009, an IAV containing a combination of segments from both the above-described triple-reassortant virus and Eurasian avian-like swine lineages spread rapidly to cause a worldwide pandemic in humans (23, 24). After the 2009 pandemic, A(H1N1)pdm09 viruses were introduced into swine populations and reassorted with endemic IAVs-S, further increasing genetic diversity among IAVs-S all over the world (25–37).

The pig industry in Japan has risen to intensive production recently; fewer but bigger pig farms are distributed around Japan, raising a total of about 9 million pigs and producing about 900,000 tons of pork in Japan in 2019. To evade the introduction of pathogens from foreign countries, imported live pigs are quarantined for 15 days at an animal quarantine station. Classical swine H1N1 IAVs-S are thought to have been introduced into the swine population in Japan during the late 1970s (38, 39). Cases of the isolation of human-like H3N2 IAVs-S from pigs were sporadic and few during 1970 to 2013 (33, 40, 41). After the introduction of a classical swine H1N1 IAV-S, it was replaced by the reassortant H1N2 IAVs-S that carried the hemagglutinin (HA) gene of the classical swine lineage and human-like neuraminidase (NA) gene. After the 1980s, the H1N2 IAVs-S have circulated predominantly among Japanese swine, and an H1N1 IAV-S possessing both H1 and N1 genes from the classical swine lineage has been rarely isolated (42–44). After the pandemic in 2009, A(H1N1)pdm09 viruses were introduced into pig populations in Japan, and genetic reassortment occurred between A(H1N1)pdm09 viruses and endemic H1N1, H1N2, and H3N2 viruses that had circulated previously (33, 41). However, information regarding IAVs-S in Japan remains sparse and localized to limited areas. Furthermore, the dynamics of IAVs-S at the farm level in Japan are unclear.

To understand the status of IAVs-S among pig populations in Japan, from 2015 through 2019, we collected nasal swab samples from pigs on farms in 21 prefectures in Japan for virus isolation and phylogenetic analyses. Simultaneously, we monitored several pig farms for at least 3 years to examine how IAVs-S circulated within these populations. The findings from our study will improve our understanding of IAVs-S currently circulating in Japan and how they evolve at the farm level.

## RESULTS

### Subtyping and geographic distribution of IAV-S in Japan.

**FIG 1:**
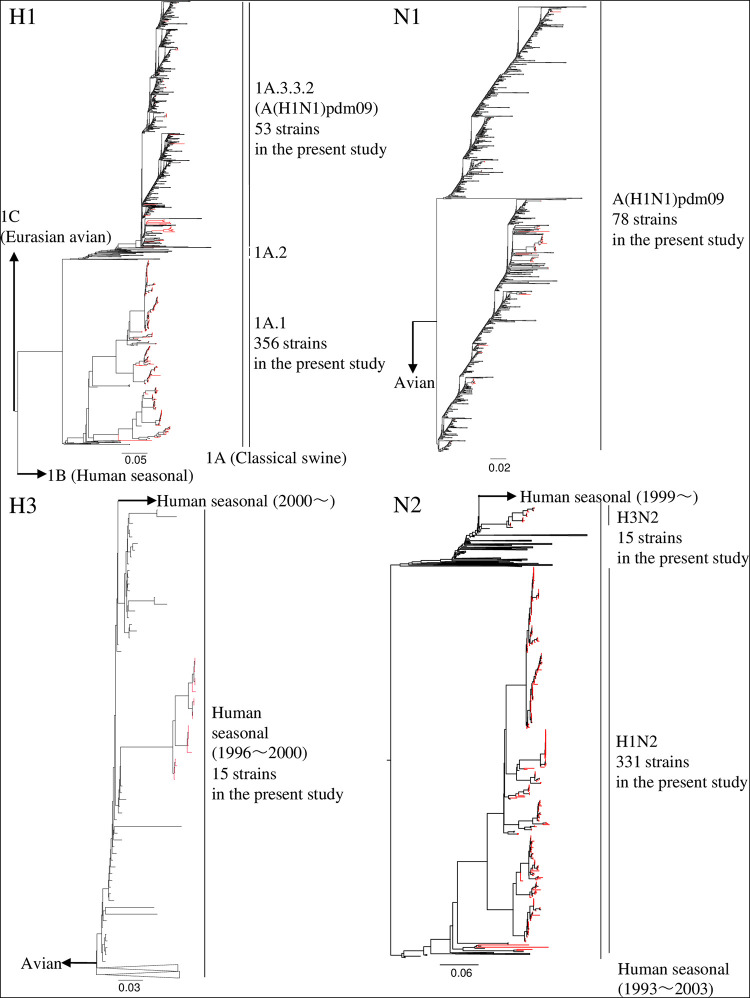
Complete maximum likelihood phylogenetic tree of the H1, H3, N1, and N2 genes from the viruses analyzed in the current study and downloaded from the GISAID databases. Red branches indicate viruses isolated in the current study.

**FIG 2:**
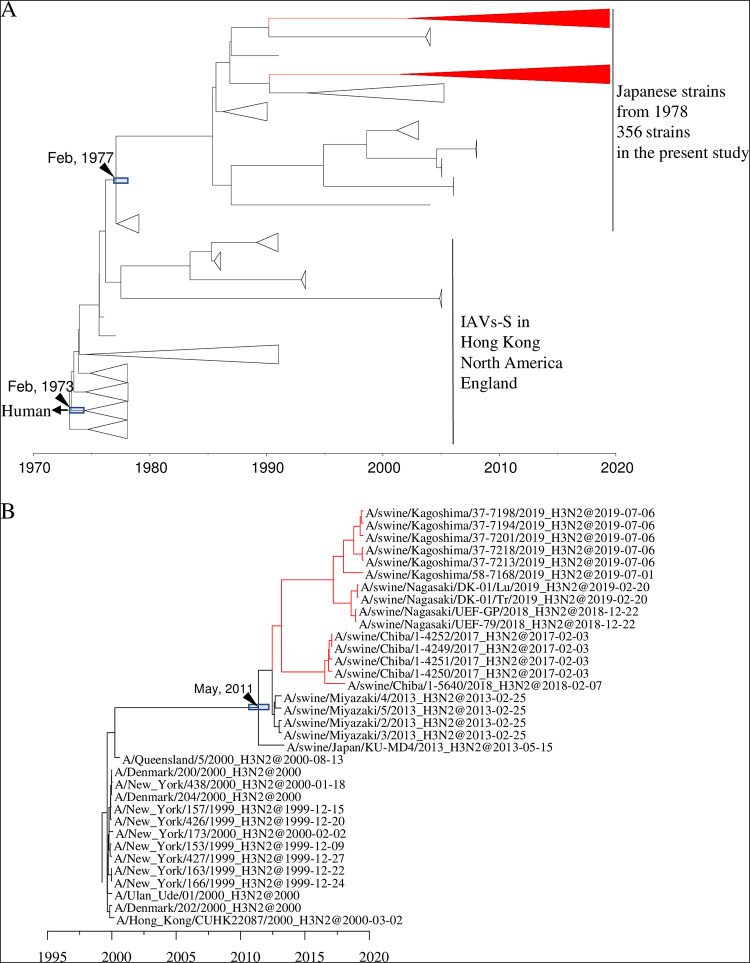
Detail of maximum clade credibility trees of H1 (A) and H3 (B) genes in the 1A.1 classical swine lineage. Red branches indicate viruses isolated in the present study. The divergence time at the branch is indicated by a black arrow, and the 95% highest posterior density for the divergence time is indicated by a gray box.

The swabs collected through the active surveillance efforts in this study yielded 370 IAVs-S, giving an overall IAV-S isolation rate of 5.2% (see Tables S1 and S2 in the supplemental material). In addition, 54 isolates were obtained from the specimens submitted for diagnosis during 2015 to 2019. Overall, 78 of the 424 total IAVs-S were subtyped as H1N1, 331 as H1N2, and 15 as H3N2. In addition, the lineages of the HA and NA genes were determined through phylogenetic analysis. Specifically, the H1 HA genes of 37 H1N1 and 319 H1N2 viruses belong to the 1A.1 classical swine lineage, whereas those of 41 H1N1 and 12 H1N2 viruses belong to the 1A.3.3.2 lineage [A(H1N1)pdm09] (Fig. 1 and Fig. S1). The H1 HA genes of the 1A.1 classical swine lineage share a common ancestor with the IAVs-S in Japan during the late 1970s, indicating that they have been circulating domestically for approximately 40 years (Fig. 2A and Fig. S2).

**FIG 3:**
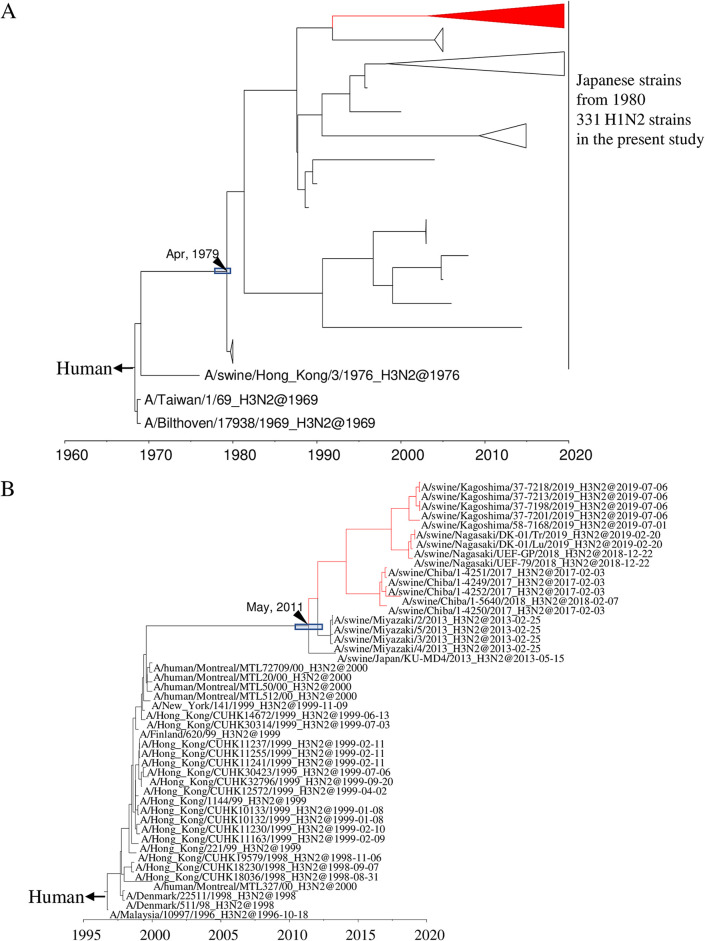
Detail of maximum clade credibility trees of N2 genes including H1N2 IAVs-S in the present study (A) and H3N2 IAVs-S in the present study (B). Red branches indicate viruses isolated in the present study. The divergence time at the branch is indicated by a black arrow, and the 95% highest posterior density for the divergence time is indicated by a gray box.

All 15 of the H3N2 IAVs-S isolated here are located in a clade belonging to the human seasonal lineage (Fig. 1 and Fig. S3). These IAVs-S are related to the human influenza viruses that circulated during the 1999–2000 season, and their ancestral strain in swine, A/swine/Japan/KU-MD4/2013, was first isolated in 2013 (Fig. 2B and Fig. S4). All 78 N1 genes of the H1N1 IAVs-S isolated here originated from A(H1N1)pdm09 viruses (Fig. 1 and Fig. S5), even though 37 of the H1N1 IAVs-S had H1 HA genes of the classical swine lineage. All 346 N2 genes of the 331 H1N2 IAVs-S and 15 H3N2 IAVs-S were classified into the human seasonal lineage (Fig. 1 and Fig. S6). However, a cluster consisting of N2 genes of the H1N2 IAVs-S was phylogenetically distinct from that of H3N2 IAVs-S. These H1N2 IAVs-S are related to the late-1960 seasonal viruses, which were derived from Hong Kong H3N2 viruses (Fig. 3A and Fig. S7), whereas those of the H3N2 IAVs-S are related to the 1999–2000 seasonal influenza viruses, which correspond to the origin of their H3 genes (Fig. 3B and Fig. S8).

The number of IAVs-S with various combinations of surface genes and the number of IAV-S-positive farms in each prefecture and year indicate the breadth of distribution of IAVs-S in Japan and their persistence on some farms (Table 1). During 2015 to 2019, H1N1 IAVs-S with solely A(H1N1)pdm09-derived surface genes (P-P) were isolated from 18 farms in 9 prefectures. H1N2 IAVs-S with 1A.1 classical swine H1 but human seasonal N2 genes (C-H) were distributed on 14 farms in 4 prefectures. Reassortant H1N1 IAVs-S, carrying 1A.1 classical swine H1 and A(H1N1)pdm09-derived N1 genes (C-P), were found on 6 farms in 2 prefectures, and those harboring 1A.3.3.2 A(H1N1)pdm09 H1 and human seasonal N2 genes (P-H) were isolated from 2 farms in a single prefecture. H3N2 IAVs-S with human seasonal H3 and N2 genes (H-H) were found on 5 farms in 4 prefectures.

**TABLE 1:** Numbers of farms positive for influenza A viruses of swine (IAVs-S) during 2015 to 2019 according to prefecture

Prefecture	2015							2016							2017							2018							2019						
	No. of positive farms (no. of strains isolated)					No. of negative farms	No. of tested farms	No. of positive farms (no. of strains isolated)					No. of negative farms	No. of tested farms	No. of positive farms (no. of strains isolated)					No. of negative farms	No. of tested farms	No. of positive farms (no. of strains isolated)					No. of negative farms	No. of tested farms	No. of positive farms (no. of strains isolated)					No. of negative farms	No. of tested farms
	H1N1		H1N2		H3N2(H-H)			H1N1		H1N2		H3N2(H-H)			H1N1		H1N2		H3N2(H-H)			H1N1		H1N2		H3N2(H-H)			H1N1		H1N2		H3N2(H-H)		
	(P-P)^*a*^	(C-P)	(P-H)	(C-H)				(P-P)	(C-P)	(P-H)	(C-H)				(P-P)	(C-P)	(P-H)	(C-H)				(P-P)	(C-P)	(P-H)	(C-H)				(P-P)	(C-P)	(P-H)	(C-H)			
Hokkaido	2 (4)					2	4	1 (1)					3	4						4	4						4	4						1	1
Tochigi																						1 (1)			1 (3)		1	3				2 (2)		0	2
Ibaraki				1 (1)		0	1																									1 (1)		0	1
Gunma	1 (1)	3 (5)		4 (13)		1	8	1 (9)	4 (5)		8 (129)		0	9				7 (51)		5	12	2 (5)			8 (26)		1	10				8 (59)		3	11
Chiba		1 (8)		3 (3)		0	3		1 (5)		1 (21)		0	1		1 (11)			1 (4)	0	1		1 (3)		1 (1)	1 (1)	0	1				1 (9)		1	1
Niigata																	1 (6)			0	1			1 (2)			0	1			2 (4)			0	2
Aichi								3 (5)					12	15								1 (1)					0	1							
Tottori																						1 (3)					0	1							
Okayama																													1 (1)					0	1
Nagasaki									1 (2)				1	2												1 (2)	0	1					1 (2)	0	1
Miyazaki								1 (2)					1	2																				2	2
Kagoshima	1 (1)					0	1						3	3	1 (1)					0	1	2 (4)					0	2					2 (6)	1	3
Aomori																																		1	1
Iwate																																		1	1
Yamagata																																		1	1
Shizuoka																																		1	1
Hiroshima																											1	1							
Fukuoka																											1	1							
Saga																																		1	1
Oita													1	1																					
Kumamoto													2	2																					

a Surface genes derived from A(H1N1)pdm09 (P), 1A.1 classical swine (C), and human seasonal (H) viruses, respectively. For example, H1N1(C-P) indicates IAVs-S with 1A.1 classical swine HA and A(H1N1)pdm09 NA genes.

**FIG 4:**
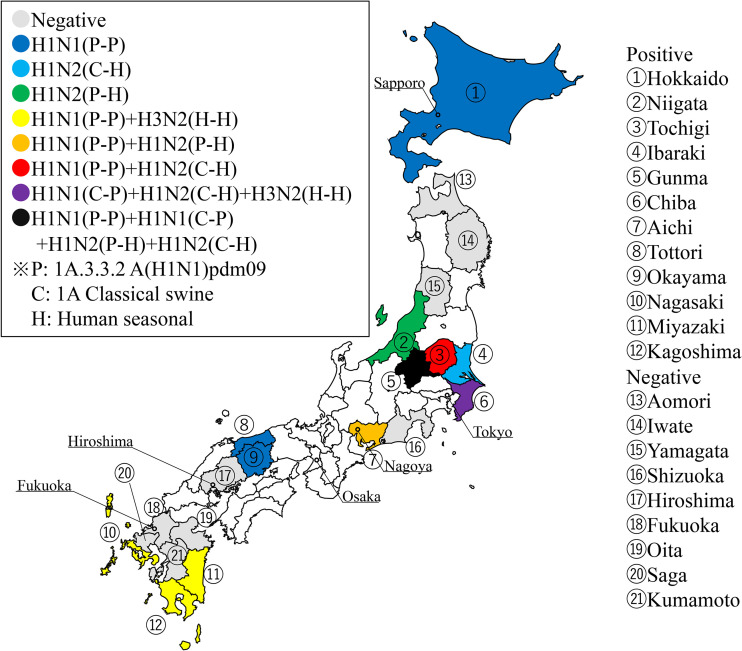
Geographic locations of prefectures where nasal swab and lung tissue samples were collected were plotted on the map (https://www.kabipan.com/geography/whitemap/; CC BY-NC 2.1 JP). Gray indicates prefectures where no IAVs-S were isolated; other colors indicate prefectures from which IAVs-S with various combinations of HA [1A3.3.2 A(H1N1)pdm09 H1, 1A.1 classical swine H1, and human seasonal H3] and NA [A(H1N1)pdm09 N1 and human seasonal N2] were isolated.

The geographic distributions of the viruses corresponding to these 5 combinations of surface genes were plotted on the map of Japan (Fig. 4). Of the 21 prefectures where samples were collected during 2015 to 2019, C-H H1N2 IAVs-S were isolated from 4 prefectures around the center of Japan (Chiba, Gunma, Ibaraki, and Tochigi). P-P H1N1 IAVs-S were isolated from 9 prefectures: 3 on the southern island, Kyushu (Kagoshima, Miyazaki, and Nagasaki); 5 on the main island, Honshu (Aichi, Gunma, Okayama, Tochigi, and Tottori); and 1 on the northern island, Hokkaido. C-P H1N1 IAVs-S were isolated in Chiba prefecture, and P-H H1N2 IAVs-S were obtained in Niigata and Aichi prefectures. H-H H3N2 IAVs-S were isolated from 3 prefectures on Kyushu (Kagoshima, Miyazaki, and Nagasaki) and from Chiba prefecture.

**FIG 5:**
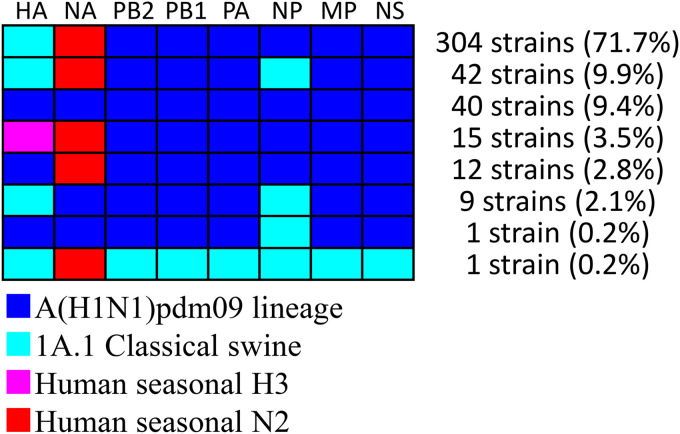
Genetic constellations revealed by phylogenetic analyses. Genes classified as A(H1N1)pdm09, 1A.1 classical swine, and human seasonal H3 and N2 were colored with blue, light blue, purple, and red, respectively. Detection rate according to the total number of IAVs-S (424 strains) for each combination is shown under the bar.

Gene constellations and their numbers in the present study revealed all IAVs-S isolated here had at least 1 gene that was derived from an A(H1N1)pdm09 virus, except for a single strain isolated in Tochigi prefecture: A/swine/Tochigi/38-7119/2019 (H1N2) inherited all of its genes from Japanese pig isolates that circulated during the 1990s and 2000s (Fig. 5 and Fig. S9).

### Genetic evolution of IAVs-S among pig populations on selected farms.

**FIG 6:**
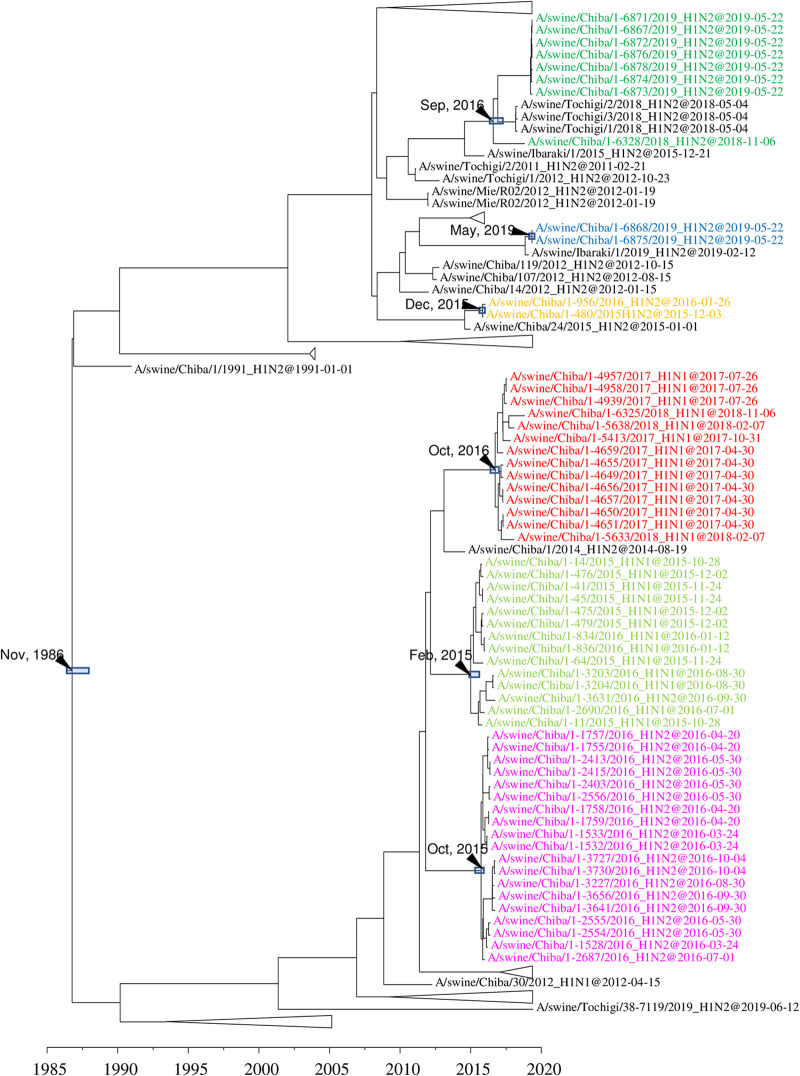
Detail of maximum clade credibility tree of H1 HA genes in the 1A.1 classical swine lineage. Colored text indicates H1N1 and H1N2 IAVs-S isolated at farm 001.

**FIG 7:**
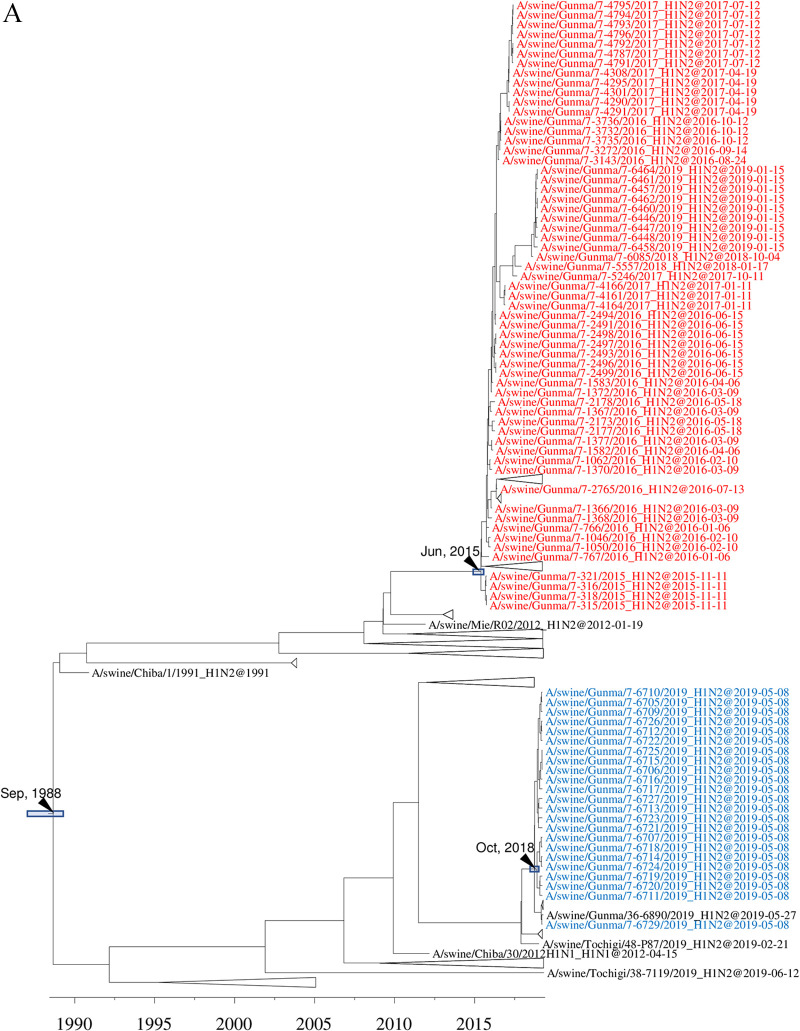

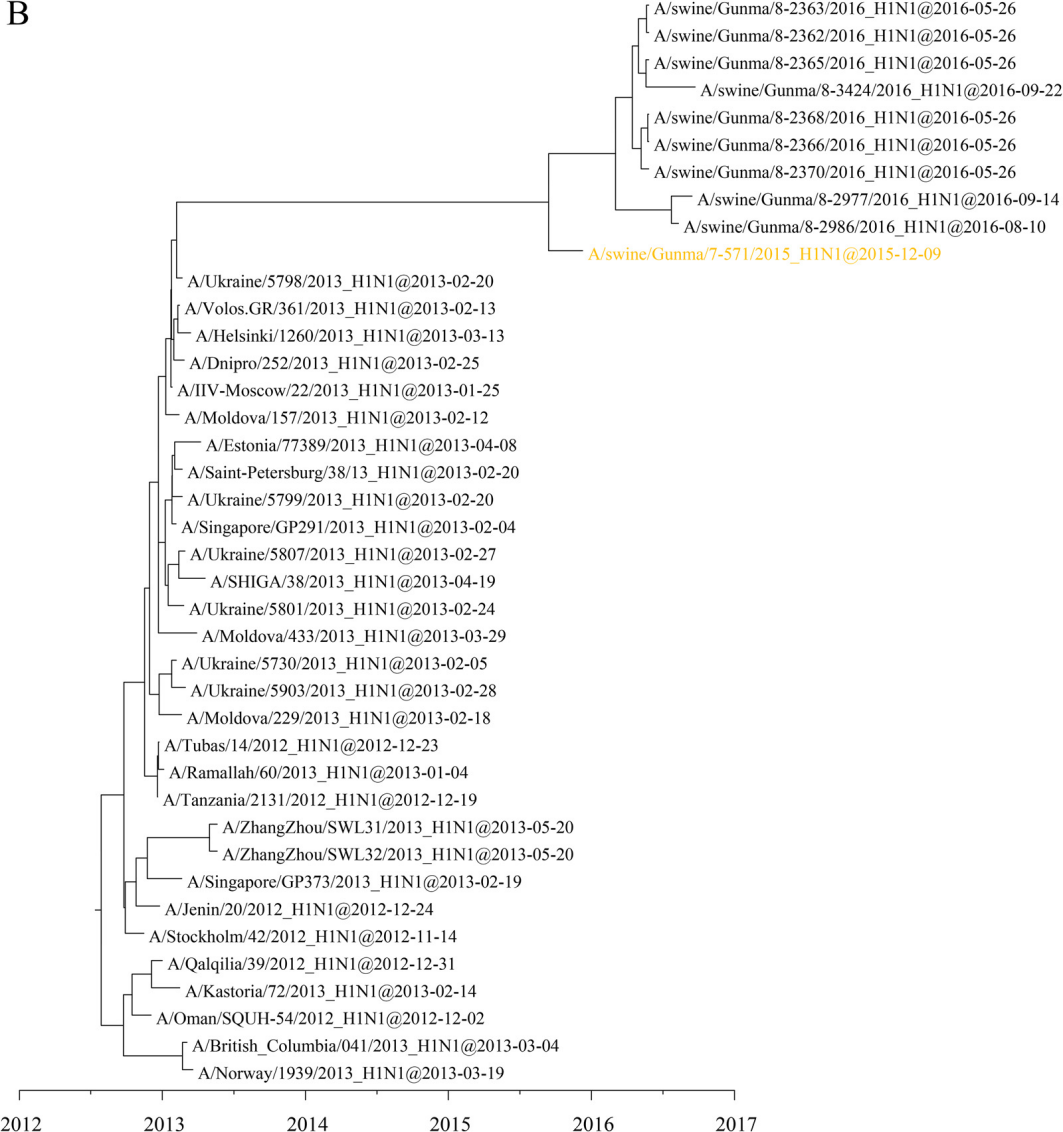
Detail of maximum clade credibility trees of H1 HA genes in the 1A.1 classical swine lineage (A) and 1A.3.3.2 A(H1N1)pdm09 lineage (B). Colored text indicates H1N1 and H1N2 IAVs-S isolated at farm 007.

**FIG 8:**
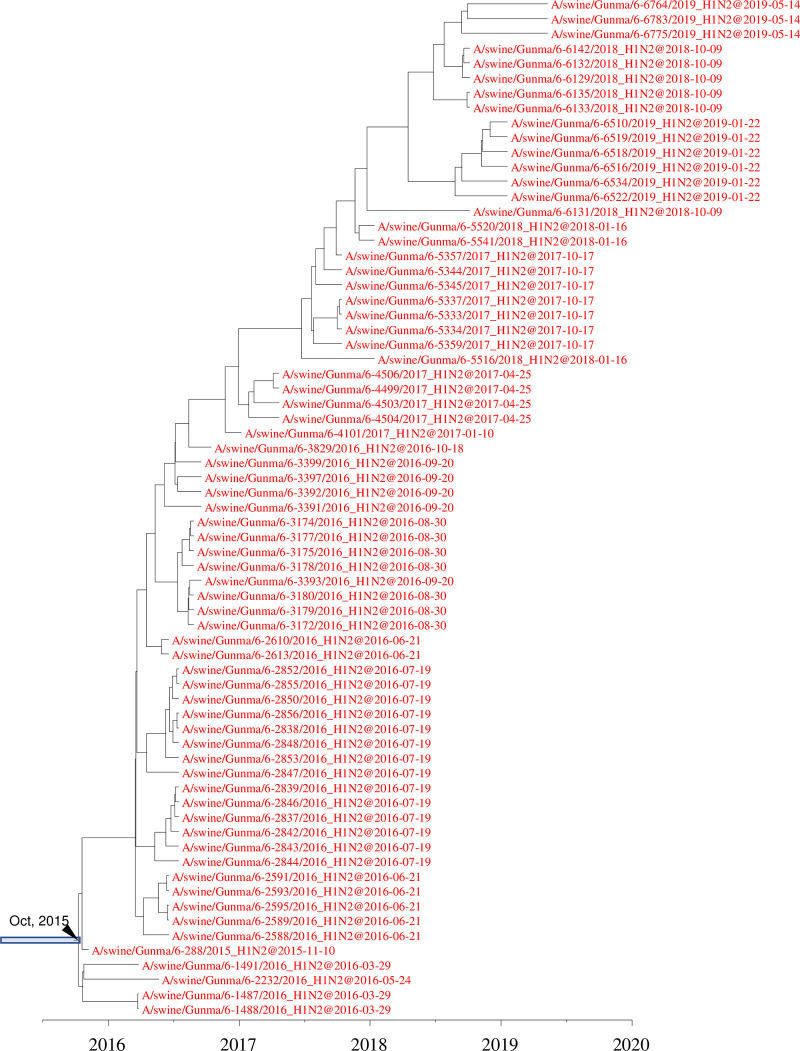
Detail of maximum clade credibility tree of H1 HA genes in the 1A.1 classical swine lineage. Red indicates H1N2 IAVs-S isolated at farm 006.

**FIG 9:**
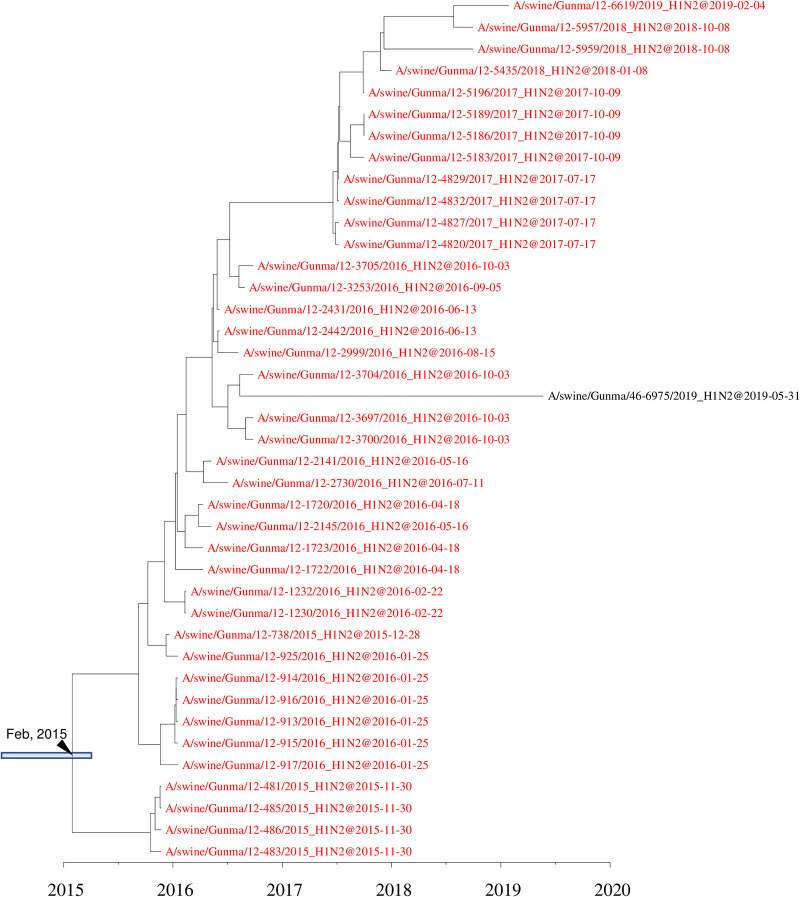
Detail of maximum clade credibility tree of H1 HA genes in the 1A.1 classical swine lineage. Red indicates H1N2 IAVs-S isolated at farm 012.

During 2015 to 2019, active surveillance over prolonged periods yielded continuous isolation of IAVs-S on some farms (Table 2). Out of the 12 farms where IAVs-S were isolated twice or more through active surveillance, single-genotype IAV-S was introduced and persisted at 4 farms (004, 034, 006, and 012), while 8 farms (001, 007, 009, 010, 011, 008, 035, and 040) experienced multiple introductions. In particular, farms 001, 006, 007, and 012 were IAV-S positive at least 15 times during 3 years or more. The evolutionary analyses of the HA genes of those isolates as well as their genetic constellations revealed that farms 001 (Fig. 6) and 007 (Fig. 7) had experienced multiple introductions of different genotypes, whereas farms 006 (Fig. 8) and 012 (Fig. 9) had virus persistence, where viruses originated from a single introduction persisting for 3 or more years. All 4 farms applied farrow-to-finish operating systems, and 300, 500, 1,000, and 2,000 sows were raised at farms 001, 006, 007, and 012, respectively.

**TABLE 2:** Results of active surveillance activity on farms where samples were collected at least twice

Prefecture	Farm no.	No. of samplings (no. IAVs-S positive)						No. of IAVs-S-positive/total no. of samplings (%)	No. of IAVs-S isolated	Duration of IAV-S persistence^*a*^ (no. of mo)	Deduced no. of introductions
		Total	2015	2016	2017	2018	2019				
Gunma	007	20 (20)	2 (2)	10 (10)	4 (4)	2 (2)	2 (2)	100	84	42	1 H1N1, 2 H1N2
Chiba	001	27 (20)	5 (4)	12 (9)	6 (4)	2 (2)	2 (1)	74.1	67	42	6 H1N2, 1 H3N2
Gunma	012	20 (16)	2 (2)	10 (9)	4 (2)	2 (2)	2 (1)	80	40	39	1 H1N2
Gunma	006	20 (15)	2 (1)	10 (7)	4 (3)	2 (2)	2 (2)	75	69	42	1 H1N2
Gunma	010	13 (9)	2 (1)	10 (8)	1 (0)	0	0	69.2	16	9	2 H1N2
Gunma	040	12 (9)	0	3 (2)	5 (4)	2 (2)	2 (1)	75	13	34	2 H1N1, 2 H1N2
Gunma	009	20 (8)	2 (1)	10 (5)	4 (1)	2 (1)	2 (0)	40	19	25	1 H1N1, 2 H1N2
Gunma	011	13 (6)	2 (1)	10 (5)	1 (1)	0	0	46.2	12	11	2 H1N2
Gunma	034	7 (5)	0	0	3 (2)	2 (1)	2 (2)	71.4	8	24	1 H1N2
Gunma	008	13 (4)	2 (0)	10 (4)	1 (0)	0	0	30.8	20	4	1 H1N1, 1 H1N2
Gunma	035	7 (4)	0	0	3 (1)	2 (2)	2 (1)	57.1	11	24	2 H1N2
Hokkaido	004	6 (2)	1 (1)	2 (1)	2 (0)	1 (0)	0	33.3	2	12	1 H1N1
Kagoshima	043	2 (1)	0	0	0	2 (1)	0	50	3		
Gunma	036	7 (1)	0	0	3 (0)	2 (0)	2 (1)	14.3	4		
Hokkaido	003	6 (1)	1 (1)	2 (0)	2 (0)	1 (0)	0	16.7	3		
Kagoshima	037	3 (1)	0	0	1 (0)	2 (1)	0	33.3	2		
Gunma	045	2 (1)	0	0	1 (0)	1 (1)	0	50	1		
Hokkaido	002	6 (0)	1 (0)	2 (0)	2 (0)	1 (0)	0	0	0		
Hokkaido	005	6 (0)	1 (0)	2 (0)	2 (0)	1 (0)	0	0	0		
Kagoshima	013	4 (0)	0	4 (0)	0	0	0	0	0		
Kagoshima	027	2 (0)	0	2 (0)	0	0	0	0	0		

a The longest period that IAV-S was repeatedly isolated in the farm, regardless of genotypes.

On farm 001, H1N1 and its reassortant H1N2 IAVs-S (yellow-green) were circulating from October 2015 to September 2016 (Fig. 6). Phylogenetically distinct H1N2 IAVs-S (orange) were sporadically isolated during the same period. Closely related but apparently distinct H1N2 IAVs-S (purple) emerged on the farm and circulated from March to October 2016; this was followed by replacement by other related H1N1 strains (red) from April 2017 to November 2018. In February 2017, H3N2 IAVs-S were isolated at the farm, and a progeny IAV-S was isolated 1 year later (Fig. 2B and Fig. S4). Introduction of two other clusters of H1N2 IAVs-S occurred during 2018 to 2019. One cluster has been isolated since November 2018 (green), and another was first isolated in May 2019 (blue). The N2 genes of A/swine/Chiba/1-6328/2018 and 7 IAVs-S isolated in May 2019 were genetically distinguishable from the N2 genes of other H1N2 IAVs-S at the farm (Fig. S7); the N2 genes of these viruses were phylogenetically distinct from those of the H3N2 IAVs-S. All internal genes of the H1N2 isolates were derived from A(H1N1)pdm09 viruses. In contrast, 5 of the internal genes (PB2, PB1, PA, MP, and NS) of the H1N1 IAVs-S were derived from A(H1N1)pdm09, but all of the NP genes were derived from 1A.1 classical swine H1 IAVs-S closely related to Japanese pig isolates during the 1990s and 2000s (Fig. 5 and Fig. S9). All of the internal genes of the H3N2 IAVs-S at the farm were A(H1N1)pdm09 in origin. However, the PB2, PB1, PA, and MP genes were phylogenetically distinguishable from those of H1 IAVs-S on the farm, although NP and NS genes were shared with them, indicating the reassortment of H1 and H3 IAVs-S.

In the case of farm 007, two phylogenetically distinct 1A.1 classical swine H1 IAVs-S were introduced (Fig. 7A and B). One was first isolated in November 2015 and persisted until January 2019; the other was isolated in May 2019. In addition to 1A.1 classical swine H1 IAVs-S, an 1A.3.3.2 H1 IAV-S that originated from an A(H1N1)pdm09 virus circulating within the human population during the 2012–2013 season was isolated in December 2015. Likewise, the NA genes of those isolates were phylogenetically classified into 3 groups: 2 groups derived from human seasonal-derived N2 genes and the remaining group from an A(H1N1)pdm09-derived N1 gene. All internal genes were derived from A(H1N1)pdm09 viruses and comprised 3 phylogenetically distinct groups.

At two farms, 006 and 012, H1N2 IAVs-S with 1A.1 classical swine H1 and human seasonal N2 genes derived from a single introduction had circulated for more than 3 years (Fig. 8 and 9). On farm 006, H1N2 IAVs-S were first isolated in November 2015, and they persisted until May 2019. At farm 012, H1N2 IAVs-S persisted from November 2015 to February 2019. An IAV-S genetically related to the strains on farm 012, A/swine/Gunma/46-6975/2019, was isolated at another farm in the same prefecture during May 2019. The N2 and internal genes of all IAVs-S isolated at farm 006 formed a single clade in phylogenetic trees constructed for all genes, indicating that they were all derived from the single introduction. Likewise, all genes of the IAVs-S at farm 012 formed a single clade.

## DISCUSSION

The aims of this study were to illustrate the current situation of IAVs-S in pig populations in Japan and to show how IAVs-S genetically evolved at farms where they were isolated over a long period of time. Our phylogenetic analyses of HA genes revealed that 3 types of IAVs-S have been isolated in Japan. As reported previously (42–44), the 1A.1 classical swine H1 lineage in Japan has evolved uniquely in Japanese pig populations, as neither a human nor foreign isolate carrying an HA gene related to 1A.1 classical swine H1 genes of Japanese pig isolates has been reported since the late 1970s. Although triple-reassortant H3N2 viruses related to isolates in Canada were obtained in Japan during animal quarantine (45), they do not appear to have become introduced into the swine population in Japan. Instead, multiple introductions of 1A.3.3.2 A(H1N1)pdm09 viruses from humans have affected the Japanese swine population. As has been reported throughout the world, A(H1N1)pdm09 viruses repeatedly became introduced into swine in Japan and then reassorted with endemic IAVs-S (25–37). Frequent introductions of 1A.3.3.2 A(H1N1)pdm09 viruses occurred on Hokkaido, mainland Japan, and Kyushu. Consequently, 1A.3.3.2 A(H1N1)pdm09 viruses that became introduced into the swine population and reassorted with Japanese endemic H1N2 IAVs-S were observed in Niigata prefecture. All cases of the isolation of 1A.3.3.2 A(H1N1)pdm09 viruses were sporadic, except for those from farms in Niigata prefecture, where H1N2 IAVs-S possessing 1A.3.3.2 A(H1N1)pdm09 HA genes persisted from May 2017 to May 2019. In a previous study, experimental infection of pigs with 5 IAVs-S of 3 subtypes (H1N1, H1N2, and H3N2), followed by cohousing, yielded H1N2 IAVs-S that were more viable than other subtypes (46). Although the epidemiologic and genetic background in the previous experiment differed from those in our current study, circulation in the pig population in Niigata prefecture might have selected H1N2 IAVs-S with increased stability and viability.

IAVs-S with an H3 HA gene of human seasonal strains have been reported sporadically in Japan (33, 40, 41), but the current situation differs from that during 1970 to 2013. H3N2 IAVs-S derived from human seasonal influenza viruses were isolated in Hokkaido in 1985, Ehime in 2002, and Osaka in 2007 (47). However, no descendant strains have been isolated subsequently, suggesting that these H3N2 IAVs-S have disappeared without becoming established within the Japanese pig population. In contrast, the remaining H3N2 IAVs-S that inherited the HA gene from the human 1999–2000 seasonal lineage, including those we collected in our current study, were isolated in 2013 (40) and from diagnostic specimens submitted from Miyazaki prefecture, Kyushu. Subsequent strains carrying HAs of the same lineage were isolated in 2017, 2018, and 2019 in Kyushu and Chiba prefectures (Fig. 2B). This finding suggests that H3N2 IAVs-S harboring the earlier-mentioned lineage of the HA gene adapted to swine well enough to become established within the Japanese pig population. One possible mechanism of this adaptation is that these H3N2 IAVs-S acquired internal genes derived from A(H1N1)pdm09 viruses. A previous study demonstrated that an avian H9N2 influenza virus adapted better to pigs when the internal genes were replaced by those from A(H1N1)pdm09 viruses (48). This possibility should be further explored concerning the H3N2 IAVs-S currently circulating in Japan.

Vaccination is one of the most frequently implemented strategies to control IAV-S on farms. However, the vaccine will be ineffective when the vaccine strain and field strains differ in antigenicity; when mutations occur at antigenic sites of the HA gene in a field strain of IAV-S that is of the same lineage as a vaccine strain; or when a virus possessing HA of a different lineage from the vaccine strain circulates in the field (49). The vaccine strains available in Japan were derived from isolates in the 1960s to 1970s, which are phylogenetically distinct from IAVs-S currently circulating in Japan. Therefore, vaccine strains need to be updated to combat classical swine H1, human seasonal H3, and 1A.3.3.2 A(H1N1)pdm09 and, thus, effectively control the current IAV-S situation in Japan. Further, current vaccination coverage against IAV-S of Japanese swine is, at most, about 10%. This is partially because swine influenza is not regarded as a critical or notifiable disease in Japan.

Multiple introductions of IAVs-S with the same subtype but different lineages of the HA genes might have contributed to the long-term isolation of IAVs-S on farms 001 and 007, as we previously suggested (37). The level of immunity to a particular strain of IAV-S against experimentally infected IAVs-S might be high enough to hamper reinfection for approximately 100 days (50). If the same was true for farm levels, the herd immunity achieved against preexisting IAVs-S could suppress the prevalence of preexisting IAVs-S for several months. However, it could not prevent a newly introduced IAV-S, providing that the newly introduced virus expresses an HA protein of different antigenicity from that of the preexisting one. This mechanism might be a factor in the long-term isolation of IAVs-S from a farm, such as farms 001 and 007; however, the occurrence of substitutions that are considered to influence the antigenicity of the HA proteins at positions 155 (51, 52) and 185 (53) could be another persistence-associated factor. On farm 012, glutamic acid 155 (Sa site) in the HA protein was shared by IAVs-S from November 2015 to July 2016 and then was replaced by glycine in the IAVs-S collected from October 2016 to February 2019. Likewise, along with one amino acid substitution at the antigenic site (168 on Ca1 site), aspartic acid at position 185 (Sb site) in the HA protein was shared among IAVs-S from November 2015 to April 2017 and then was replaced by asparagine in the IAVs-S from October 2017 to May 2019 on farm 006 (see Table S3 in the supplemental material). Single mutations at positions 145, 155, 156, 158, 159, 189, and 193 that are adjacent to the receptor binding site of the human H3 HA protein greatly reduced reactivity against serum obtained from ferrets infected with a wild-type strain (54). Of these 7 mutations, the single mutation at position 145 on H3 HA protein among IAVs-S also reduced reactivity against serum obtained from pigs infected with a wild-type strain (55). Likewise, in the present study, the single mutation at position 155 on H1 HA protein, which corresponds to 158 on H3 HA protein, might be critical for the altered antigenicity, enabling the variant to evade immunity against the previous strain on the farm. However, demonstration of an effect of lineage replacement and substitution at the antigenic site on the antigenicity of the HA protein is necessary to prove this hypothesis.

In summary, our phylogenetic analysis reveals the current situation of IAVs-S in Japan and the genetic evolution of IAVs-S on various farms. Whereas the 1A.1 classical swine H1 IAVs-S and human seasonal H3 IAVs-S have become established among pig populations in Japan, 1A.3.3.2 A(H1N1)pdm09 IAVs-S have become introduced repeatedly from the human population. Therefore, IAVs-S surveillance should be continued to understand the status of IAVs-S among the pig population in Japan and to support vaccine strategies compatible with the current situation in Japan.

## MATERIALS AND METHODS

### Sample collection.

Through active surveillance efforts during 2015 through 2019, we collected 7,133 nasal swabs from pigs in various age groups at farms in Japan. During the same period, nasal swab samples and lung tissue samples collected from pigs suspected to have swine influenza were transferred from prefectural animal hygiene centers to the National Institute of Animal Health, Japan, for diagnosis. Through active and passive surveillance, we collected samples from 21 of all 47 prefectures. Nasal swabs collected through active surveillance were put promptly into minimum essential medium containing penicillin (1,000 U/ml), streptomycin (1,000 μg/ml), amphotericin B (25 μg/ml), HEPES (0.01 M), and 0.5% bovine serum albumin and were kept on ice packs until transport to the National Institute of Animal Health, Japan. There, the swabs were removed and the medium aliquoted and stored at –80°C until virus isolation. From each sample, 200 μl was used for RNA extraction and virus detection.

### Virus detection and isolation.

RNA was extracted from swab samples by using RNeasy minikits (Qiagen, Hilden, Germany), as described previously (32, 56). The extracted RNA was reverse transcribed to cDNA by using Superscript III (Invitrogen, Carlsbad, CA, USA) and universal primers for influenza A virus (57). This cDNA was the template for real-time PCR analysis using SYBR premix Ex Taq (TaKaRa Bio, Shiga, Japan) with primers specific for the matrix protein (MP) gene, as described previously (58), or the MP gene was detected using an AgPath-ID one-step reverse transcription-PCR kit (Applied Biosystems, Foster City, CA, USA) with primers and probes according to the diagnostic manual of the CSIRO Australian Animal Health Laboratory (59, 60). For virus isolation, media from nasal swabs that were virus positive according to real-time PCR analysis were filtered (pore size, 0.45 μm; Millipore, Danvers, MA, USA) and inoculated into cultures of floating MDCK cells, 9- to 11-day-old embryonated chicken eggs, or primary cultures of porcine alveolar epithelial cells, as previously reported (47, 61). The supernatants of these cell cultures were used in HA assays (68) with guinea pig erythrocytes.

### Genomic sequencing and phylogenetic analysis.

All gene segments of isolated viruses were sequenced by using next-generation sequencing; consensus sequences were generated and the HA and NA genes of each isolate were subtyped by using FluGAS software as described previously (37). Briefly, the output paired-end reads from the MiSeq second-generation sequencer (Illumina, San Diego, CA, USA) were mapped to reference sequences that were selected from a search of the Influenza Virus Database of the National Center for Biotechnology Information (NCBI) by using the FluGAS algorithm; this was followed by the construction of a consensus sequence when at least 3 reads were available. A single nucleotide was adopted when its representation exceeded 51% at that site, whereas mixed-base codes were adopted when multiple bases each accounted for at least 15% of the total coverage at the site. The nucleotide sequences of the IAVs-S analyzed here have been deposited in the GISAID EpiFlu database; isolate identifier numbers are listed in Table S4 in the supplemental material. For phylogenetic analyses, all H1 and H3 HA genes and N1 and N2 NA genes in the GISAID database were downloaded in September 2019. The sequences of the IAVs-S isolated here and the downloaded sequences from GISAID were aligned by using BioEdit (62) and MAFFT (63). Maximum likelihood trees based on aligned sequences (H1, 55,345; H3, 68,720; N1, 56,555; and N2, 70,938 strains) were constructed by using FastTree, version 2.1.10 (64), and clusters containing Japanese IAVs-S with fast-global bootstrap values of 90 or greater were further analyzed.

To calculate molecular estimates of divergence times from ancestral IAVs for selected clusters containing HA and NA genes of IAVs-S, maximum clade credibility trees were constructed as previously reported (65).

Sequences for the polymerase basic protein 1 (PB1) genes of IAVs isolated from humans (55,504 strains) and swine (5,793 strains) were downloaded from the GISAID database, and nonredundant sets of human and swine sequences were produced by using CD-HIT software (66) at an identity threshold of 99.5%. Other gene segments of strains selected as described earlier (human, 1,475 strains; swine, 2,056 strains) were used for constructing maximum likelihood trees. A/swine/Gunma/10-1636/2016 was removed from the analysis, because several gene segments included many mixed bases, indicating that this isolate can be considered a mixture of two IAVs-S. Tanglegrams were constructed from the maximum likelihood trees for individual genes by using Dendroscope 3 (67).

### Data availability.

See Table S4 for a complete list of the EpiFlu accession numbers of the IAVs-S in the present study.

## Supplementary Material

Supplemental file 1

Supplemental file 2

Supplemental file 3

Supplemental file 4

Supplemental file 5
