# Supplementary material for: Genetic Characterization of Influenza A Viruses in Japanese Swine in 2015 to 2019
Source: J Virol. 2020 Jul 1;94(14):e02169-19. doi: 10.1128/JVI.02169-19 (PMC7343197; doi:10.1128/JVI.02169-19)
Supplement: Supplemental file 1 [file JVI.02169-19-s0001.pdf]

Figure S1
H1

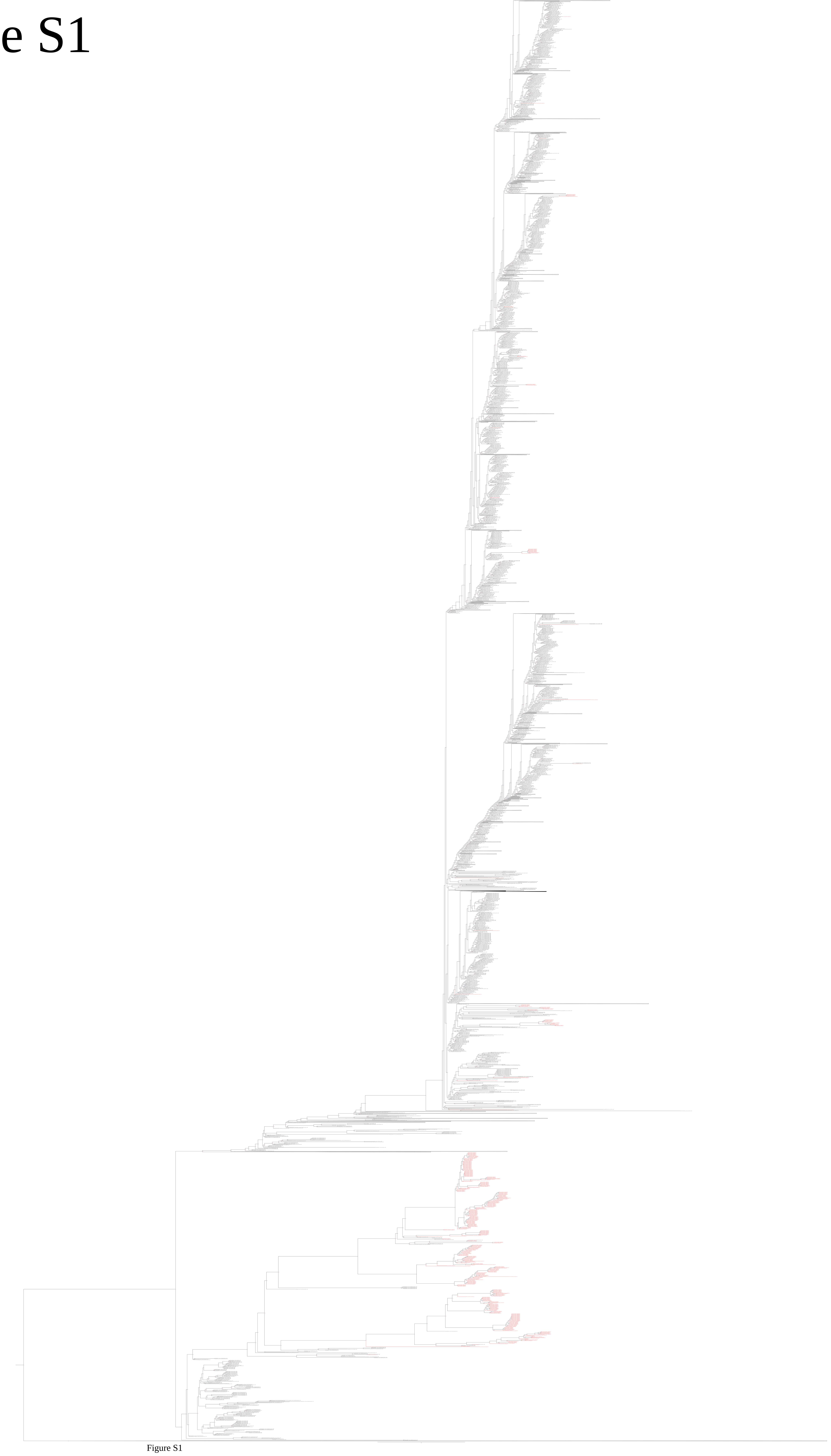

Figure S1

Complete maximum likelihood phylogenetic tree of the H1 genes from the viruses analyzed in the current study and downloaded from the GISAID databases. Red branches indicate viruses isolated in the current study. Red values indicate the high fast-global bootstrap values and blue values indicate the low fast-global bootstrap values.

Figure S2
H1 Classical swine

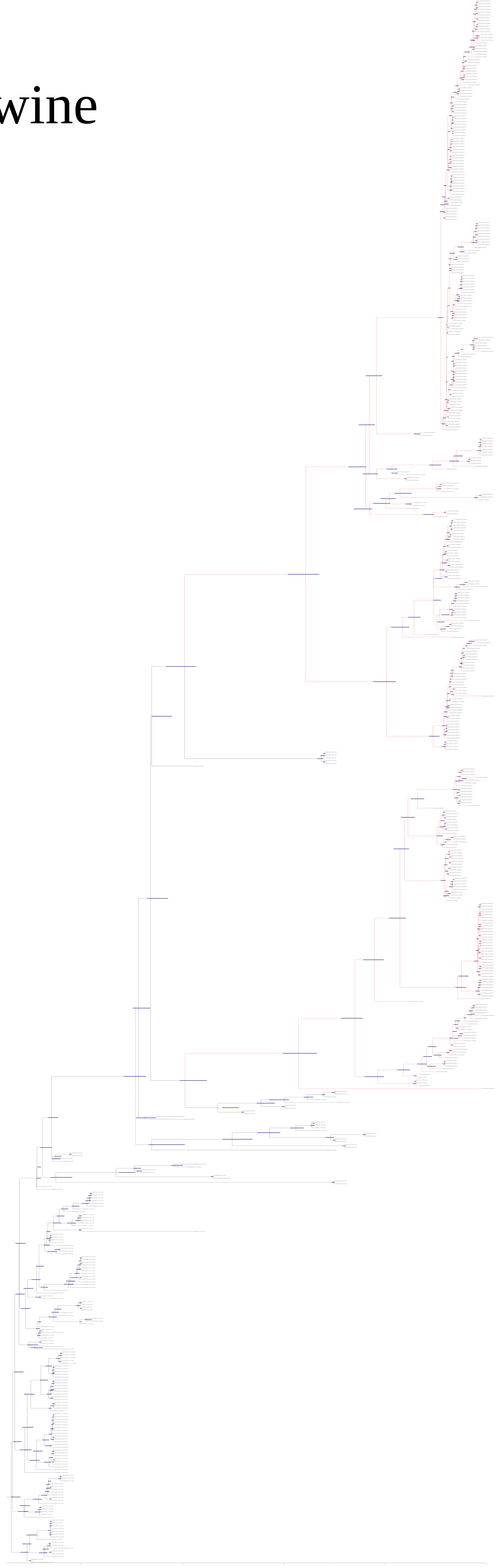

Figure S2

Detail of maximum clade credibility trees of H1 genes in the 1A.1 classical swine lineage. Red branches indicate viruses isolated in the present study. The divergence time at the branch is indicated and the 95% highest posterior density for the divergence time is indicated by a gray box.

Figure S3
H3

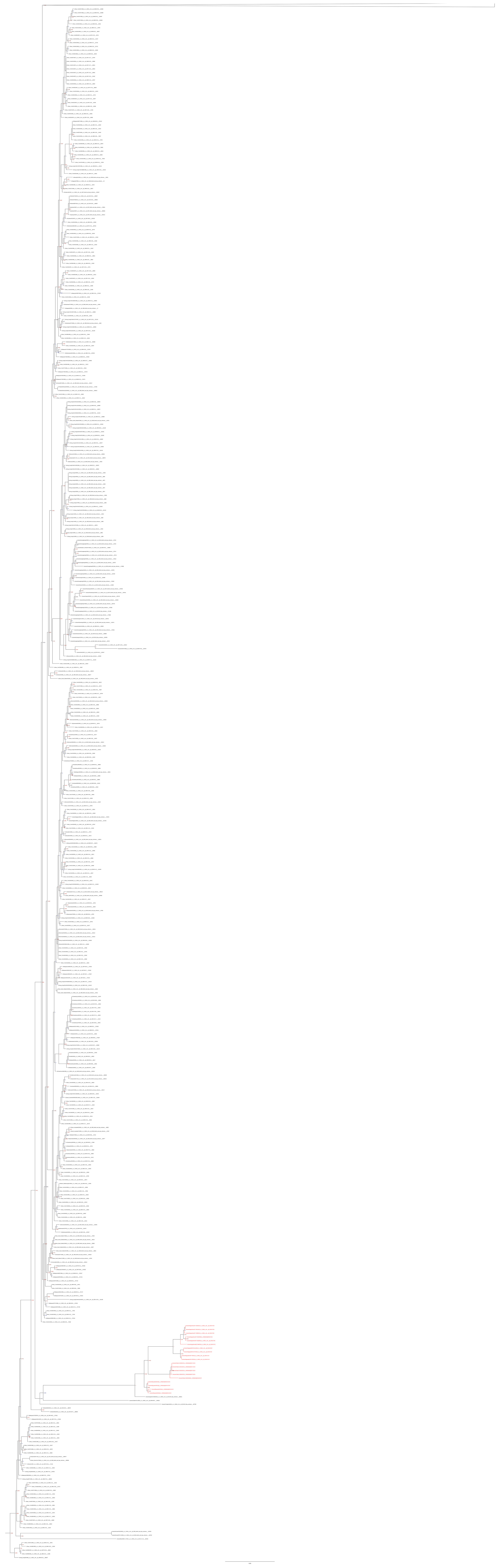

Figure S3

Complete maximum likelihood phylogenetic tree of the H3 genes from the viruses analyzed in the current study and downloaded from the GISAID databases. Red branches indicate viruses isolated in the current study. Red values indicate the high fast-global bootstrap values and blue values indicate the low fast-global bootstrap values.

Figure S4
H3

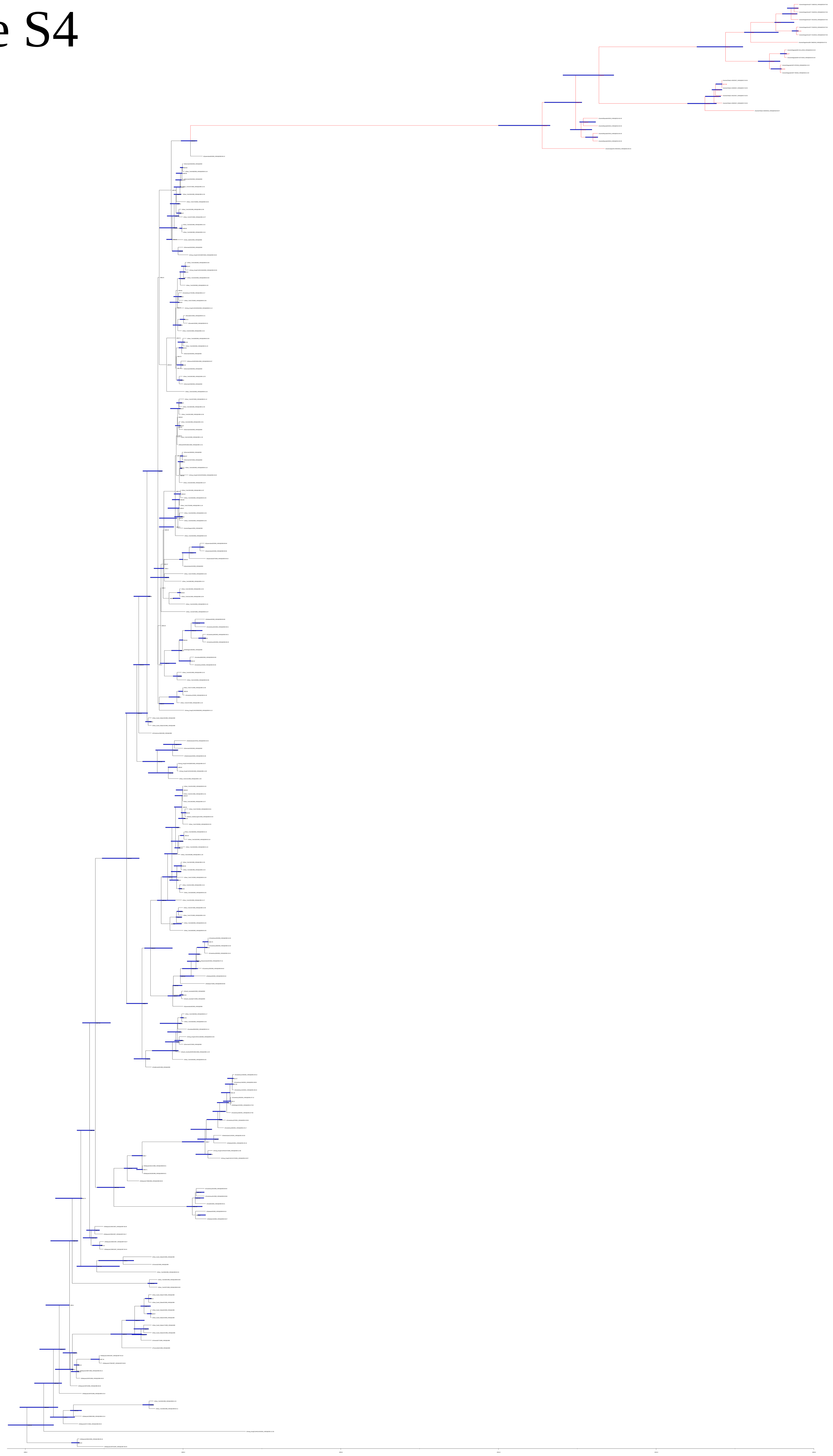

Figure S4

Detail of maximum clade credibility trees of H3 genes. Red branches indicate viruses isolated in the present study. The divergence time at the branch is indicated and the 95% highest posterior density for the divergence time is indicated by a gray box.

Figure S5
N1

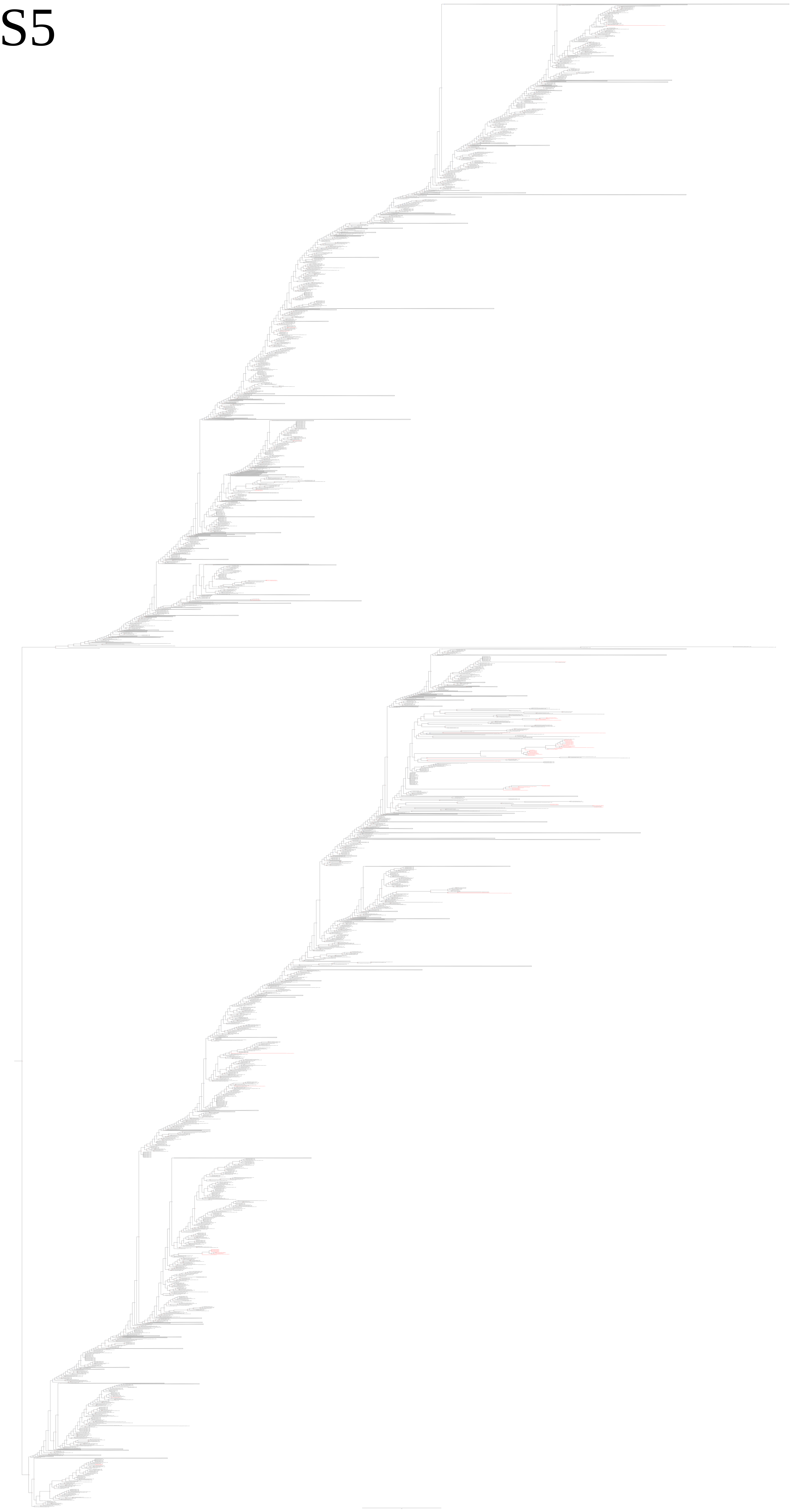

Figure S5

Complete maximum likelihood phylogenetic tree of the N1 genes from the viruses analyzed in the current study and downloaded from the GISAID databases. Red branches indicate viruses isolated in the current study. Red values indicate the high fast-global bootstrap values and blue values indicate the low fast-global bootstrap values.

Figure S6
N2

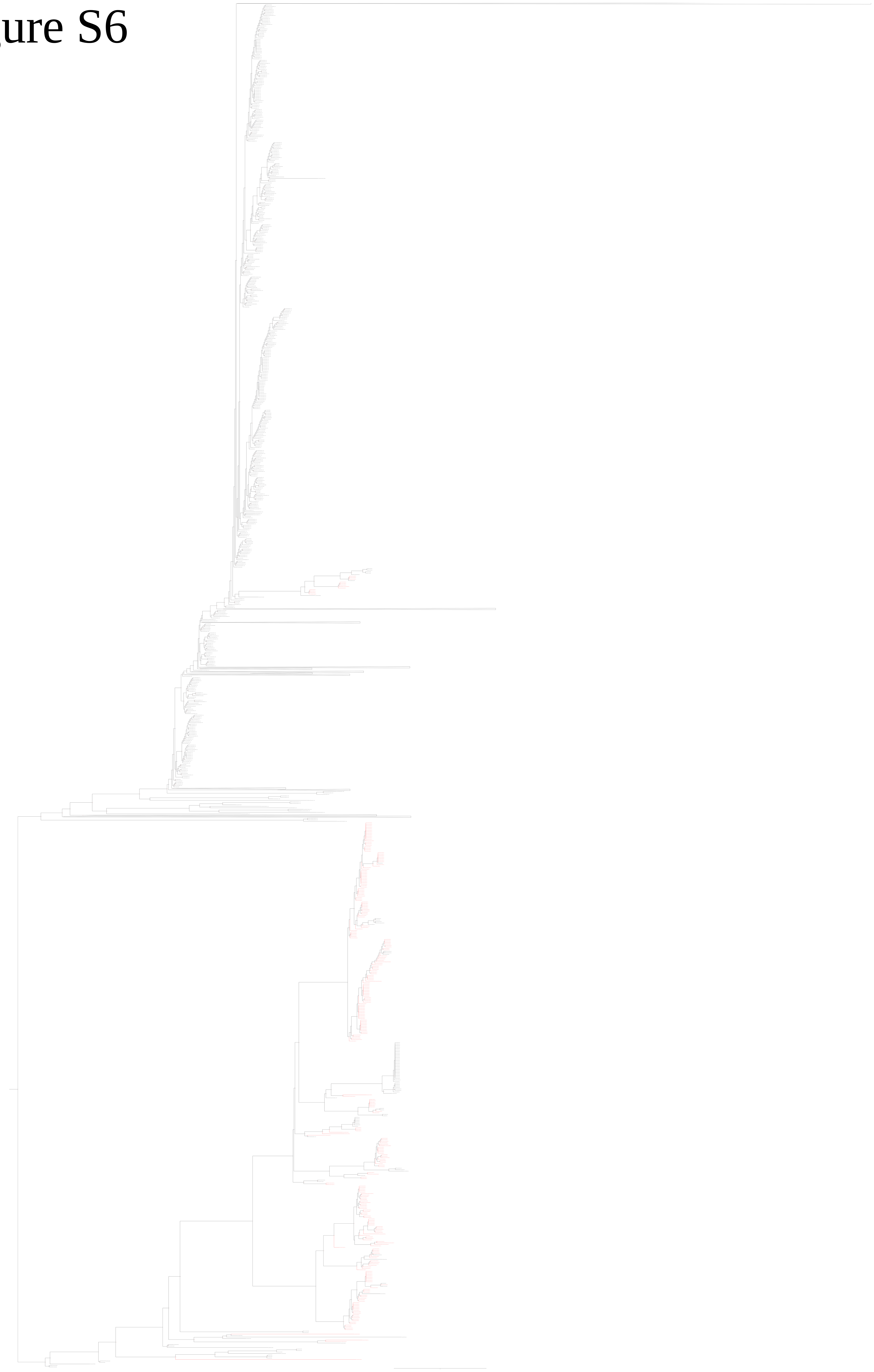

Figure S6

Complete maximum likelihood phylogenetic tree of the N2 genes from the viruses analyzed in the current study and downloaded from the GISAID databases. Red branches indicate viruses isolated in the current study. Red values indicate the high fast-global bootstrap values and blue values indicate the low fast-global bootstrap values.

Figure S7
N2(H1N2)

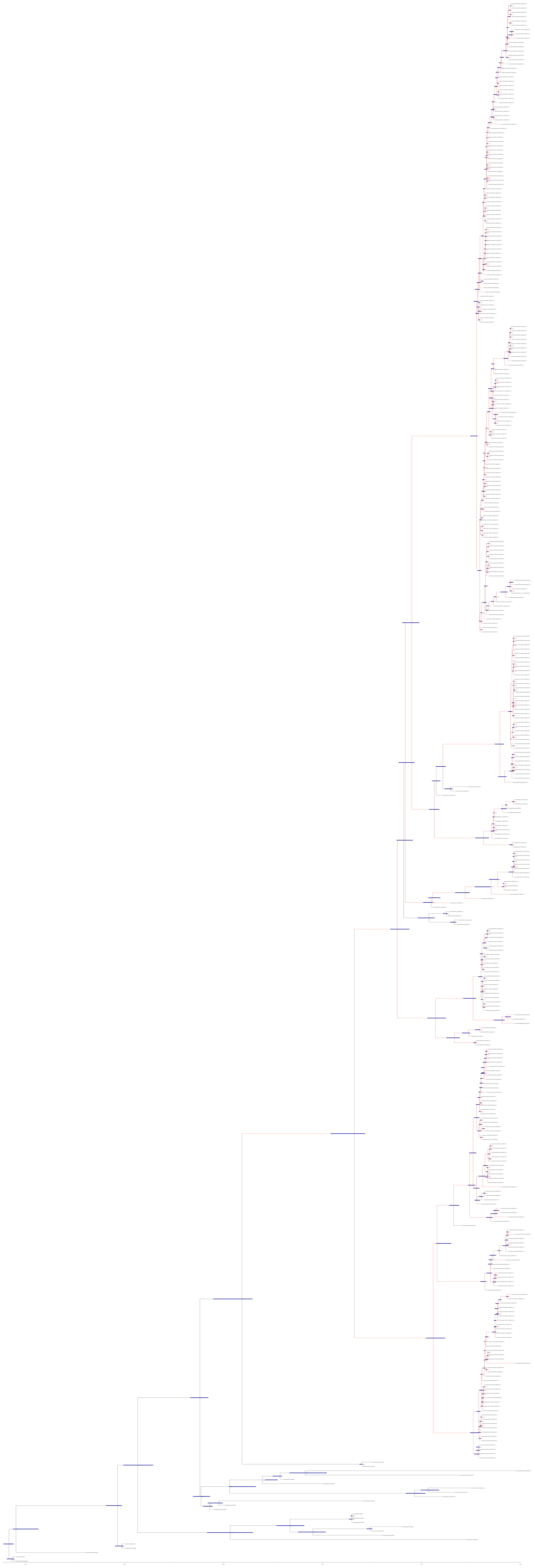

Figure S7

Detail of maximum clade credibility trees of N2 genes including H1N2 IAVs-S. Red branches indicate viruses isolated in the present study. The divergence time at the branch is indicated and the 95% highest posterior density for the divergence time is indicated by a gray box.

Figure S8

N2(H3N2)

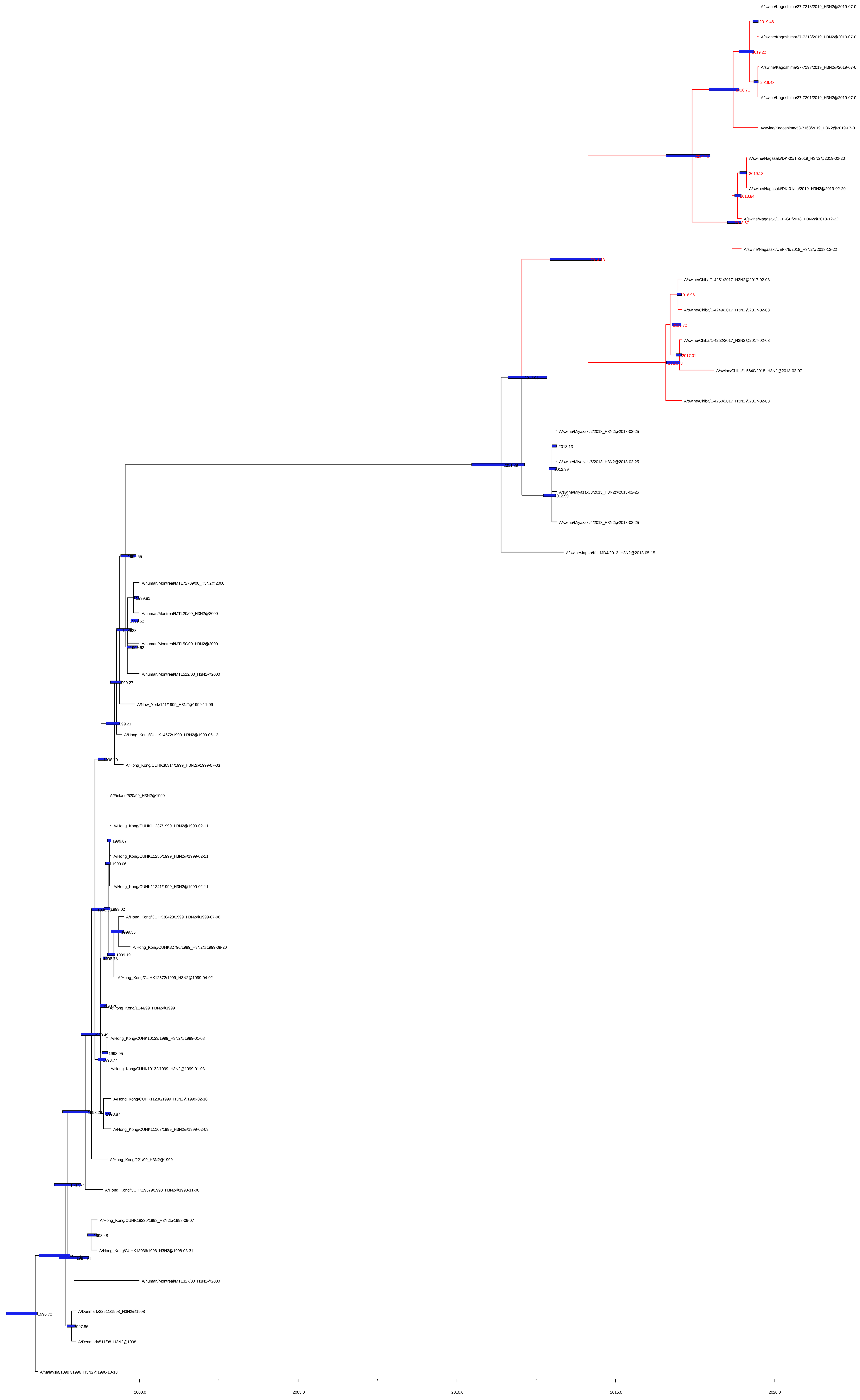

Figure S8

Detail of maximum clade credibility trees of N2 genes including H3N2 IAVs-S. Red branches indicate viruses isolated in the present study. The divergence time at the branch is indicated and the 95% highest posterior density for the divergence time is indicated by a gray box.

Figure S9

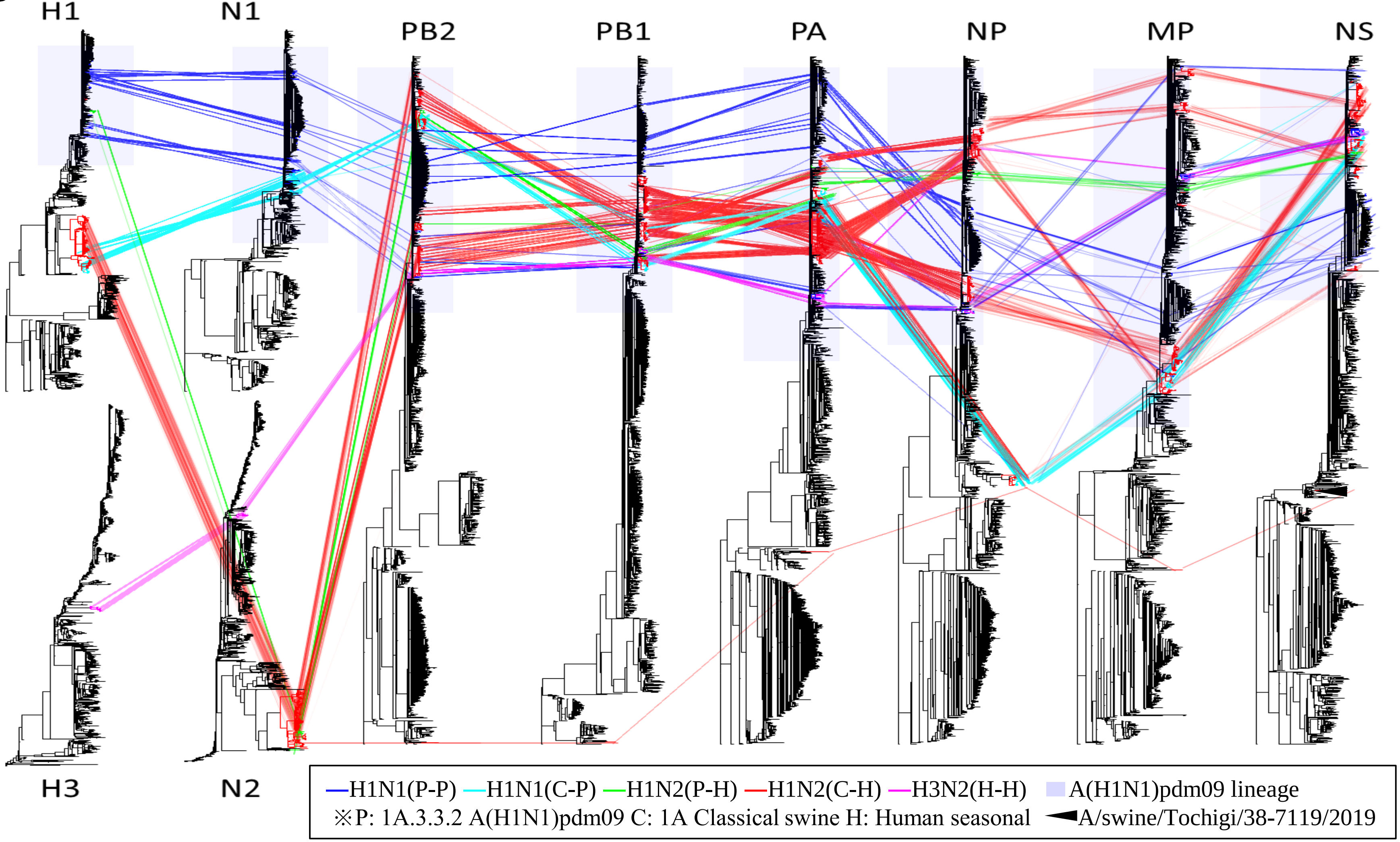

Figure S9

Tanglegrams constructed from the various maximum likelihood phylogenetic trees. Corresponding taxa in adjacent trees are connected by lines. Colors of branches and lines are: cyan, H1N1 IAVs-S with A(H1N1)pdm09-derived H1 and N1 genes (P-P); light blue, H1N1 IAVs-S carrying 1A.1 classical swine H1 and A(H1N1)pdm09-derived N1 genes (C-P); green, H1N2 IAVs-S with A(H1N1)pdm09-derived H1 and human seasonal N2 genes (P-H); red, H1N2 IAVs-S harboring 1A.1 classical swine H1 and human seasonal N2 genes (C-H); and purple, H3N2 IAVs-S carrying human seasonal H3 and N2 genes (H-H).
